# Is the different time trend (1997–2008) of the obesity prevalence among adults in the three Belgian regions associated with lifestyle changes?

**DOI:** 10.1186/2049-3258-72-18

**Published:** 2014-06-02

**Authors:** Sabine Drieskens, Johan Van der Heyden, Stefaan Demarest, Jean Tafforeau

**Affiliations:** 1 Scientific Institute of Public Health, Operational Direction Public Health and Surveillance, Unit Surveys, Lifestyle and Chronic Conditions, Brussels, Belgium

**Keywords:** Health Interview Survey, Obesity, Lifestyle, Physical activity, Eating habits, Smoking, Alcohol drinking, Time trend, Health promotion, Belgium

## Abstract

**Background:**

Obesity is a major public health issue with increasing prevalence among adults. However, in Belgium the regional time trends (1997–2008) differed: the prevalence of obesity increased in the Flemish and Brussels Regions, but remained stable in the Walloon Region, the latter still showing the highest prevalence. The purpose of the present study is to explore if the different time trends of obesity prevalence in the three Belgian regions is associated with lifestyle changes.

**Methods:**

We used data from four successive cross-sectional waves (1997, 2001, 2004 and 2008) of the Belgian Health Interview Survey. The study was restricted to the adult population, resulting in samples of respectively 8,071, 9,391, 10,319 and 8,831 individuals. In line with the WHO definition, obesity was defined as having a BMI ≥ 30. Differences in regional trends of obesity were investigated through stratified analyses. The association between obesity and survey year, adjusted for lifestyle factors (alcohol consumption, smoking, fruit and vegetables consumption and leisure time physical activity), was assessed via logistic regression models. Interactions were added to the models to explore if the association between lifestyle factors and obesity varied over time.

**Results:**

Obesity was associated with daily alcohol use in the Brussels (OR 0.66, 95% CI 0.50-0.88) and Walloon Regions (OR 0.8, 95% CI 0.6-0.9), with lower tendencies of being obese for daily drinkers. The probability of being obese was lower among smokers in the Flemish (OR 0.7, 95% CI 0.6-0.8) and Walloon Regions (OR 0.7, 95% CI 0.6-0.9) than among non-smokers. A lack of leisure time physical activity was associated with the probability of being obese in all regions (Brussels Region: OR 1.6, 95% CI 1.3-1.8; Flemish Region: OR 1.6, 95% CI 1.4-1.9; Walloon Region: OR 1.8, 95% CT 1.6-2.1). This association decreased significantly between 1997 and 2008 only in the Walloon Region.

**Conclusion:**

The decreasing association between obesity and a lack of leisure time physical activity in the Walloon Region between 1997 and 2008 could indicate that there is an increasing awareness of risk factors for obesity in the Walloon population, which may have resulted in a more favourable evolution of the obesity epidemic.

## Background

Obesity is a major public health issue, not only because it has a negative impact on the quality of life [1-3], but also because it is an important risk factor for serious chronic conditions (cardiovascular diseases, type 2 diabetes, some cancers,…) and premature deaths [1,2,4,5]. According to the World Health Organization (WHO), obesity is an ‘escalating epidemic’ [6]. Over the past decades, the prevalence of obesity among adults has increased in both developed and developing countries [1,7-12].

This was also the case for Belgium. Results from the Belgian Health Interview Surveys (HIS) revealed a linear increase of almost 28% of the national prevalence of obesity between 1997 and 2008 [13]. This national increase concealed important regional disparities. The Flemish and Brussels Regions shared a similar increase of obesity, whereas the Walloon Region remained stable at the relatively high level. This is particularly interesting, because each region has its own public health authority managing the health promotion and preventive care, although linking this to the different regional trends in obesity is not based on evidence but on speculations.

Obesity results from an imbalance between dietary intakes and energy expenditure through physical activity [14,15]. When there is a higher calorie intake through food or drinks than is burned, then the energy balance leans towards a weight gain. The WHO has identified counteracting obesity as one of the priority areas of public health action [16]. Obesity is a serious risk factor, which is largely preventable through lifestyle changes [17].

Unhealthy diet and physical inactivity, have been identified as causative factors of obesity, and are themselves consequences of social changes and economic development [12,18]. Unhealthy diet habits are partly due to an overconsumption of cheap energy dense food, eating more frequently, dining out, increased portion size (in restaurants and food packages), but also to the rising cost of healthy foods compared to unhealthy foods [11,19]. Simultaneously, the introduction of new technologies has influenced people’s occupation and daily activities with a decrease of physical activities. In addition, people rely more on motorised transport and spend more of their leisure time doing sedentary activities, such as watching television, playing video games and using the computer [11,18,20]. Studies show a positive association between obesity and leisure time physical activity: the prevalence of obesity declines as the amount of leisure time physical activity increases [21-23].

In addition to diet [3,5,19] and physical activity [3,5,19,23,24], alcohol consumption and tobacco use [2] could also be lifestyle factors that would impact the prevalence of obesity. The high energy content of alcohol makes its consumption a potential contributor. A high consumption of alcohol (more than 3 alcoholic drinks per day, with one drink being equivalent to 10 g of ethanol) is associated with the risk of abdominal obesity in men [25]. Contrarily, smoking and obesity are inversely related. Although the body weight among smokers is lower than among non-smokers, heavy smokers are more obese than moderate smokers, probably as a consequence of other risk behaviours, such as low physical activity, poor diet and a greater alcohol intake [26,27].

The purpose of the present study is to explore if the different time trends of obesity prevalence in the three Belgian regions is associated with lifestyle changes. More specifically, it will be assessed if the different time trends are still observed after adjustment for the four selected lifestyle factors and if their association with obesity has changed over time in all regions.

## Methods

The Belgian Health Interview Survey (HIS) is a periodical cross-sectional study, having been conducted in 1997, 2001, 2004 and 2008. The fieldwork of the fifth survey began in 2013. A representative sample of the Belgian population was selected including all persons residing in Belgium with no restrictions on age or nationality. Quarterly updates of the National population Registry were used as the sampling frame. A multistage clustered sampling design was applied involving a geographical stratification, a selection of municipalities within provinces, of households within municipalities, and of respondents within households. Using matched substitution of non-participating households assured the creation of the predefined net-sample size and composition. The basic methodology has been described by Demarest et al. [28]. The overall response rate varied between 55% and 61% [13].

Data were collected through a face-to-face interview using PAPI (Paper and Pencil Interviewing) at the participant’s home. The interviews were supplemented with a self-administered questionnaire (for the participants aged 15+) covering more sensitive topics including mental health, alcohol consumption and sexual behaviour [29]. The questionnaires included existing validated instruments when possible. The survey was carried out in line with the Belgian privacy legislation and approved by an ethical committee.

The Body Mass Index (BMI = kg/m^2^) is the most commonly used index of relative weight among adults [1,8]. The analysis in this study was restricted to adults aged 18 years and older. According to the recommendation of the WHO, adult individuals, both women and men, with a BMI ≥ 30 were considered as obese [6]. The study population consisted of 8,071, 9,391, 10,319 and 8,831 individuals in respectively 1997, 2001, 2004 and 2008. Questions on height and weight were included in the face-to-face questionnaire with the following wording: ‘How tall are you without shoes? (cm)’ and ‘How much do you weight without clothes and shoes? (Kg)’. Pregnant women were asked to report their weight before pregnancy.

The four lifestyle factors selected in this study were based on their relevance and the availability of the data in the HIS during the four surveyed years. For the statistical analysis, these lifestyle factors were recoded as dichotomous indicators:

1. ‘daily alcohol drinking’ (AL) was in 1997, 2001 and 2004 derived from 4 questions, whereby a subdivision between weekdays and weekend days was made (validated WHO questions). First it was asked if people drink during weekdays. If so, the number of days was asked. The same was applied for the weekend days. This was not the case in 2008. Only one general question was asked (validated question, recommended by Eurostat): ‘During the past 12 months, how often have you had alcoholic drinks of any kind (beer, wine, cider, spirits, cocktails, premixes, liquor, homemade alcohol…)?’ , with response categories 1) Every day or almost, 2) 5–6 days a week, 3) 3–4 days a week, 4) 1–2 days a week, 5) 2–3 days in a month, 6) Once a month, 7) Less than once a month, 8) Not in the past 12 months, as I no longer drink alcohol and 9) Never, or only a few sips or trials in my whole life. The response categories were recoded in two groups: daily drinkers versus all other categories;

2. ‘current smokers’ (TA) was based on a combination of two questions: a) a filter question (only used in 2004 and 2008) ‘Have you smoked at least 100 cigarettes (about 5 packets) or the equivalent amount of tobacco in your entire life?’ (response categories: 1) yes and 2) no) and b) the validated WHO question (in 2008 slightly adapted to the guidelines of Eurostat) ‘Do you (now) smoke (at all nowadays)?’ (response categories: 1) Yes, daily, 2) Yes, occasionally and 3) Not at all). People who have smoked less than 100 cigarettes (first question) are not considered as ‘current smokers’ (no = 2), and those who have smoked that amount or more are considered as ever-smokers. ‘Current smokers’ (yes = 1) are those who smoke daily and those who smoke occasionally (response categories 1 and 2 of the second question) among the ever-smokers;

3. ‘daily consumption of fruit and vegetables’ (NH) was derived from a food frequency questionnaire (validated instrument in 2001, but adapted) and combines the results from two questions: ‘How often do you eat fruit?’ and ‘How often do you eat vegetables?’ Respondents indicating ‘once a day’ for both questions are considered as ‘daily consumers’ , all others as ‘not daily consumers’;

4. ‘lack of leisure time physical activity’ (PA ) was based on a question recommended by a WHO group of experts [30] ‘What best describes your leisure time activities during the last year?’ , with the answer categories (two additional categories added to the validated instrument): 1) Hard training and competitive sport more than once a week, 2) Jogging and other recreational sports or gardening, at least 4 hours per week, 3) Jogging and other recreational sports or gardening, at most 4 hours per week, 4) Walking, bicycling or other light activities at least 4 hours a week, 5) Walking, bicycling or other light activities at most 4 hours a week and 6) Reading, watching TV or other sedentary activities, taking the last answer category (yes, lack = 1) versus the other answer categories (no lack = 2).

The questions on nutritional habits were included in the face-to-face questionnaire while the questions on the use of alcohol and tobacco were included in the self-administered questionnaire. The question on leisure time physical activity was first included in the face-to-face questionnaire (1997 and 2001) and later moved to the self-administered questionnaire (2004 and 2008).

Detailed information on educational attainment was collected from the face-to-face questionnaire and recoded into four educational categories, as proposed by the Organisation for Economic Co-operation and Development (OECD) [31]: no or primary education, secondary inferior, secondary superior and tertiary education. Educational attainment was assessed at household level and defined as the highest educational degree of the reference person or his/her partner.

The evolution over time of obesity in the three regions was assessed by using a logistic regression to calculate the Odds Ratio (OR) and the 95% confidence intervals (95% CI) of being obese, adjusted for age, gender and education (proxy for socio-economic status, a potential confounder [1,2,32-35]), with the Belgian population of 2001 as reference. A description of these (background) variables was given through the crude prevalence.

The effect of the selected lifestyle factors was first explored by presenting crude prevalence by year and region. Logistic regression, stratified by region, allowed examining if adjustment for lifestyle factors added to a model with age, gender, education and year could explain the different regional trends. In a next step interactions between year and lifestyle factors were separately added to the complete model. If a significant interaction was found, this was further explored through models including the specific lifestyle indicator, age, gender and education, and stratified by year and region.

All the analyses were performed with SAS® 9.2 [36] and the SURVEYLOGISTIC procedure was used to take into account the complex survey design (weighting, clustering and stratification). As item non-response was limited, a complete-case analysis was performed.

## Results

Table 1 describes the distribution of the crude prevalence of the background variables (age, gender, educational level, year and region). This table also shows that the prevalence of obesity significantly increased up to the age group 55–64 years (OR 6.7, 95% CI 5.0-9.1), with decreasing educational level (OR that is 2.6 higher, 95% CI 2.2-3.0 for those with no or primary education than for those with a tertiary education) and from the first survey in 1997 (reference) to the fourth in 2008 (OR 1.3, 95% CI 1.1-1.5). Furthermore, a significant higher prevalence of obesity was observed in the Walloon Region (OR 1.3, 95% CI 1.2-1.5) compared to the Flemish Region. Adding the interaction year*region to this model reveals that the evolution in the Walloon Region (P = 0.0367) was significantly different from the one in the Flemish Region: while the prevalence of obesity remained stable in the Walloon Region, an increase could be observed in the Flemish Region.

**Table 1:** **Crude prevalence** (**95**% **CI**) **and odds of being obese** (**95**% **CI**), **HIS**, **Belgium 1997**–**2008**

**Variables**		**% (95% CI)**	**Odds(95% CI)**
Age group	18-24 years	3.4 (2.5-4.3)	1
	25-34 years	6.6 (5.8-7.4)	2.177 (1.589-2.982)
	35-44 years	11.9 (10.8-12.9)	4.105 (3.039-5.544)
	45-54 years	14.6 (13.4-15.8)	4.946 (3.686-6.637)
	55-64 years	20.1 (18.5-21.6)	6.733 (4.988-9.090)
	65-74 years	17.9 (16.3-19.6)	5.227 (3.846-7.105)
	75 + years	13.4 (11.7-15.0)	3.365 (2.442-4.638)
Gender	Males	11.9 (11.3-12.6)	1
	Females	12.8 (12.2-13.5)	1.063 (0.974-1.160)
Education	No or primary education	19.0 (17.5-20.5)	2.581 (2.226-2.992)
	Secondary inferior	16.8 (15.5-18.1)	2.273 (1.974-2.616)
	Secondary superior	12.6 (11.7-13.5)	1.797 (1.580-2.044)
	Tertiary education	7.4 (6.8-8.1)	1
Year	1997	10.8 (9.8-11.9)	1
	2001	12.1 (11.2-13.0)	1.067 (0.926-1.229)
	2004	12.7 (11.7-13.6)	1.155 (1.001-1.033)
	2008	13.8 (12.7-14.8)	1.307 (1.131-1.512)
Region	Flemish Region	11.5 (10.8-12.2)	1
	Brussels Region	11.1 (10.4-11.9)	1.065 (0.956-1.186)
	Walloon Region	14.5 (13.7-15.3)	1.329 (1.205-1.466)

Table 2 presents the distribution of the crude prevalence of the lifestyle factors. In summary, for all but daily drinking alcohol, the selected lifestyle factors seem to improve over time in every region.

**Table 2:** **Crude prevalence** (**95**% **CI**) **by lifestyle factor**^&^, **year and region**, **HIS**, **Belgium 1997**–**2008**

	**Year**	**Flemish Region**	**Brussels Region**	**Walloon Region**
AL	1997	6.5 (5.3 – 7.7)	8.6 (7.1 – 10.1)	9.6 (7.7 – 11.4)
	2001	8.5 (7.4 – 9.6)	13.0 (11.3 – 14.7)	10.6 (9.2 – 12.0)
	2004	8.1 (7.0 – 9.3)	11.7 (10.1 – 13.3)	10.6 (9.1 – 12.1)
	2008	11.3 (9.8 – 12.8)	11.3 (9.7 – 12.9)	13.6 (11.8 – 15.5)
TA	1997	24.1 (21.9 - 26.2)	27.0 (24.5 – 29.6)	27.5 (25.1 – 29.9)
	2001	22.9 (21.1 – 24.7)	25.4 (23.2 – 27.6)	26.0 (24.0 – 28.0)
	2004	22.6 (20.6 – 24.6)	23.8 (21.4 – 26.1)	25.7 (23.5 – 27.9)
	2008	18.7 (16.7 – 20.6)	22.3 (20.0 – 24.6)	24.0 (21.6 – 26.4)
NH	1997	22.2 (20.0 – 24.4)	20.3 (17.9 – 22.6)	27.4 (24.9 – 29.9)
	2001	46.9 (44.5 – 49.2)	36.3 (33.8 – 38.8)	39.8 (37.4 – 42.1)
	2004	44.9 (42.6 – 47.2)	41.8 (39.2 – 44.4)	39.5 (37.0 – 42.0)
	2008	60.2 (57.8 – 62.6)	53.9 (51.4 – 56.4)	54.6 (52.2 – 57.1)
PA	1997	28.8 (26.4 – 31.1)	41.2 (38.2 – 44.2)	38.8 (35.9 – 41.8)
	2001	29.3 (27.2 – 31.5)	38.2 (35.8 – 40.6)	42.6 (40.2 – 44.9)
	2004	20.8 (19.0 – 22.6)	27.0 (24.6 – 29.5)	31.7 (29.3 – 34.1)
	2008	22.2 (20.1 – 24.2)	30.9 (28.3 – 33.5)	34.9 (32.3 – 37.4)

^&^Lifestyle factors: daily drinking alcohol (AL), current smoking (TA), eating daily fruit and vegetables (NH) and lack of leisure time physical activity (PA).

Table 3 shows that, after adjustment for age, gender, education and year, daily drinking alcohol was not associated with obesity in the Flemish Region, which contrasted to the findings of the Brussels and the Walloon Regions with lower odds of being obese in daily alcohol drinkers (respectively OR 0.7, 95% CI 0.5-0.9 and OR 0.8, 95% CI 0.6-0.9). The odds of being obese was significantly lower among current smokers in the Flemish (OR 0.7, 95% CI 0.6-0.8) and the Walloon Regions (OR 0.7, 95% CI 0.6-0.9), but this was not the case in the Brussels Region. Eating daily fruit and vegetables was not associated with obesity. A lack of leisure time physical activity increased significantly the odds of being obese in all regions: OR of 1.6 (95% CI 1.3-1.8) and 1.6 (95% CI 1.4-1.9) in respectively the Brussels and the Flemish Region. In the Walloon Region this OR was even 1.8 (95% CI 1.6-2.1).

**Table 3:** **Odds** (**95**% **CI**) **of being obese**, **stratified by region**, **HIS**, **Belgium 1997**–**2008**

**Region**	**Variables**		**Odds(95%CI)**
Flemish Region	Age	-	1.017 (1.013-1.021)
	Gender	Males	1
		Females	1.021 (0.893-1.168)
	Education	No or primary	1.913 (1.502-2.436)
		Secondary inferior	2.349 (1.888-2.923)
		Secondary superior	1.724 (1.415-2.101)
		Tertiary	1
	Year	-	1.038 (1.017-1.059)
	Daily drinking alcohol		0.901 (0.699-1.161)
	Current smoking		0.692 (0.577-0.829)
	Eating daily fruit and vegetables		0.956 (0.825-1.107)
	Lack of leisure time physical activity		1.631 (1.400-1.899)
Brussels Region	Age	-	1.010 (1.007-1.014)
	Gender	Males	1
		Females	0.934 (0.803-1.087)
	Education	No or primary	3.140 (2.516-3.919)
		Secondary inferior	1.603 (1.274-2.018)
		Secondary superior	1.622 (1.310-2.009)
		Tertiary	1
	Year	-	1.038 (1.016-1.061)
	Daily drinking alcohol		0.662 (0.496-0.883)
	Current smoking		0.836 (0.682-1.026)
	Eating daily fruit and vegetables		0.956 (0.810-1.129)
	Lack of leisure time physical activity		1.556 (1.313-1.844)
Walloon Region	Age	-	1.011 (1.007-1.015)
	Gender	Males	1
		Females	0.824 (0.723-0.938)
	Education	No or primary	2.085 (1.663-2.614)
		Secondary inferior	1.875 (1.530-2.298)
		Secondary superior	1.748 (1.452-2.105)
		Tertiary	1
	Year	-	1.012 (0.995-1.030)
	Daily drinking alcohol		0.749 (0.595-0.941)
	Current smoking		0.732 (0.621-0.862)
	Eating daily fruit and vegetables		1.064 (0.932-1.216)
	Lack of leisure time physical activity		1.795 (1.562-2.063)

Table 4 indicates that significant interactions were observed between year and lifestyle indicators only in the Walloon Region. This was the case for daily alcohol consumption (P = 0.0136) and leisure time physical activity (P = 0.0145). Those interactions were further illustrated in Tables 5 and 6. In the Walloon Region no significant association was found between obesity and daily drinking alcohol in the first three survey years. Conversely, in 2008 obesity was significantly less prevalent among daily alcohol users than among non-drinkers (OR 0.5, 95% CI 0.3-0.8). In all regions and almost for all surveyed years there was a strong and significant association between being obese and being physical inactive during leisure time. However, only in the Walloon Region this association dropped substantially between 1997 (OR 2.6, 95% CI 1.9-3.6) and 2008 (OR 1.4, 95% 1.1-1.8).

**Table 4:** **Obesity**: **interactions** ‘year*lifestyle indicator^&^, (**P**-**value**) **added to Table**3, **stratified by region**, **HIS**, **Belgium 1997**-**2008**

**Interaction**	**Flemish Region**	**Brussels Region**	**Walloon Region**
+ year*AL	0.2104	0.9290	0.0136
+ year*TA	0.2898	0.0543	0.3714
+ year*NH	0.3132	0.2743	0.0888
+ year*PA	0.9546	0.9102	0.0145

^&^Lifestyle indicators: daily drinking alcohol (AL), current smoking (TA), eating daily fruit and vegetables (NH) and lack of leisure time physical activity (PA).

‘*’ indicated the interaction between two variables.

**Table 5:** **Odds (95% CI) of being obese and AL**^**&**^**, stratified by region and year, HIS, Belgium 1997–2008**

**Year**	**Variables**		**Flemish Region**	**Brussels Region**	**Walloon Region**
1997	Age		1.025 (1.015-1.034)	1.012 (1.003-1.021)	1.023 (1.014-1.032)
	Gender	Males	1	1	1
		Females	0.951 (0.678-1.334)	1.367 (0.969-1.928)	0.756 (0.565-1.012)
	Education	No or primary	2.713 (1.529-4.815)	3.370 (1.862-6.099)	1.484 (0.870-2.533)
		Secondary inferior	2.042 (1.204-3.465)	2.137 (1.290-3.542)	1.355 (0.877-2.094)
		Secondary superior	2.259 (1.404-3.636)	1.308 (0.772-2.213)	1.536 (1.035-2.277)
		Tertiary	1	1	1
	AL		1.139 (0.657-1.973)	0.825 (0.336-2.025)	0.893 (0.543-1.470)
2001	Age		1.023 (1.016-1.031)	1.009 (1.002-1.015)	1.016 (1.010-1.023)
	Gender	Males	1	1	1
		Females	1.125 (0.874-1.449)	0.940 (0.708-1.246)	0.969 (0.759-1.238)
	Education	No or primary	2.256 (1.406-3.620)	2.878 (1.934-4.284)	3.229 (2.167-4.811)
		Secondary inferior	2.969 (1.944-4.534)	1.148 (0.709-1.860)	2.234 (1.519-3.286)
		Secondary superior	1.749 (1.169-2.617)	1.765 (1.177-2.647)	2.254 (1.552-3.275)
		Tertiary	1	1	1
	AL		1.085 (0.666-1.767)	0.488 (0.294-0.811)	0.892 (0.581-1.370)
2004	Age		1.022 (1.015-1.029)	1.015 (1.009-1.021)	1.014 (1.008-1.020)
	Gender	Males	1	1	1
		Females	1.141 (0.887-1.466)	1.168 (0.879-1.552)	0.887 (0.696-1.130)
	Education	No or primary	1.785 (1.173-2.718)	3.762 (2.563-5.522)	2.458 (1.617-3.736)
		Secondary inferior	2.112 (1.414-3.156)	1.878 (1.249-2.825)	2.858 (1.972-4.141)
		Secondary superior	1.462 (1.009-2.118)	1.505 (1.009-2.246)	2.226 (1.540-3.217)
		Tertiary	1	1	1
	AL		0.565 (0.344-0.930)	0.708 (0.425-1.180)	0.833 (0.533-1.254)
2008	Age		1.015 (1.008-1.022)	1.011 (1.004-1.018)	1.009 (1.003-1.016)
	Gender	Males	1	1	1
		Females	1.144 (0.900-1.453)	0.678 (0.513-0.896)	1.010 (0.794-1.284)
	Education	No or primary	1.529 (0.956-2.444)	3.401 (2.279-5.078)	2.075 (1.414-3.044)
		Secondary inferior	2.447 (1.657-3.613)	1.667 (1.066-2.608)	1.732 (1.179-2.546)
		Secondary superior	1.611 (1.139-2.279)	1.971 (1.344-2.891)	1.498 (1.082-2.072)
		Tertiary	1	1	1
	AL		0.855 (0.543-1.348)	0.728 (0.452-1.172)	0.497 (0.312-0.790)

^&^AL: daily drinking alcohol.

**Table 6:** **Odds (95% CI) of being obese and PA**^**&**^**, stratified by region and year, HIS, Belgium 1997-2008**

**Year**	**Variables**		**Flemish Region**	**Brussels Region**	**Walloon Region**
1997	Age		1.024 (1.014-1.033)	1.010 (1.001-1.020)	1.016 (1.007-1.025)
	Gender	Males	1	1	1
		Females	0.901 (0.644-1.260)	1.311 (0.926-1.856)	0.679 (0.501-0.921)
	Education	No or primary	2.483 (1.394-4.423)	3.027 (1.716-5.338)	1.226 (0.707-2.126)
		Secondary inferior	1.896 (1.118-3.214)	1.895 (1.136-3.158)	1.134 (0.729-1.764)
		Secondary superior	2.169 (1.349-3.489)	1.235 (0.732-2.081)	1.412 (0.953-2.093)
		Tertiary	1	1	1
	PA		1.632 (1.151-2.313)	1.860 (1.287-2.687)	2.627 (1.900-3.632)
2001	Age		1.022 (1.015-1.030)	1.006 (0.999-1.013)	1.014 (1.008-1.020)
	Gender	Males	1	1	1
		Females	1.091 (0.840-1.416)	0.968 (0.726-1.292)	0.922 (0.726-1.171)
	Education	No or primary	2.140 (1.330-3.446)	2.993 (2.005-4.468)	3.064 (2.048-4.584)
		Secondary inferior	2.874 (1.881-4.393)	1.188 (0.739-1.909)	2.097 (1.423-3.090)
		Secondary superior	1.752 (1.172-2.618)	1.825 (1.217-2.738)	2.132 (1.460-3.115)
		Tertiary	1	1	1
	PA		1.356 (1.027-1.791)	1.121 (0.828-1.518)	1.594 (1.241-2.047)
2004	Age		1.020 (1.013-1.027)	1.013 (1.007-1.019)	1.012 (1.006-1.018)
	Gender	Males	1	1	1
		Females	1.132 (0.880-1.456)	1.147 (0.863-1.525)	0.848 (0.667-1.079)
	Education	No or primary	1.768 (1.165-2.683)	3.638 (2.476-5.346)	2.248 (1.472-3.434)
		Secondary inferior	2.133 (1.423-3.197)	1.774 (1.167-2.699)	2.727 (1.876-3.963)
		Secondary superior	1.467 (1.012-2.127)	1.423 (0.959-2.111)	2.144 (1.494-3.075)
		Tertiary	1	1	1
	PA		1.668 (1.247-2.232)	1.904 (1.367-2.653)	1.749 (1.316-2.324)
2008	Age		1.013 (1.006-1.020)	1.009 (1.003-1.016)	1.006 (1.000-1.013)
	Gender	Males	1	1	1
		Females	1.121 (0.882-1.425)	0.666 (0.504-0.881)	1.029 (0.809-1.307)
	Education	No or primary	1.429 (0.890-2.294)	3.438 (2.303-5.131)	2.057 (1.397-3.027)
		Secondary inferior	2.345 (1.576-3.490)	1.649 (1.317-2.875)	1.694 (1.149-2.498)
		Secondary superior	1.546 (1.096-2.180)	1.946 (1.317-2.875)	1.480 (1.068-2.051)
		Tertiary	1	1	1
	PA		1.708 (1.274-2.290)	1.479 (1.053-2.078)	1.354 (1.050-1.747)

^&^PA: lack of leisure time physical activity.

## Discussion

In view of the increasing obesity problem that is reported worldwide, the stabilisation of obesity in the Walloon Region is quite remarkable. When investigating the association of lifestyle factors influencing obesity (stratified by region), the lack of leisure time physical activity appears to be the most important determinant throughout all regions. The core findings of this study are that a) in the Walloon Region, the association between leisure time physical activity and obesity has significantly decreased during the study period and b) in contrast to the Flemish and Brussels Regions, the prevalence of obesity did not significantly increase in the Walloon Region.

From our analyses it appears that the increase in prevalence of people that are physical active during leisure time cannot entirely explain the stabilisation of the obesity prevalence. An assumption could be that an improvement of leisure time physical activity among obese people in the Walloon Region could possibly also be associated with a healthier lifestyle in general. This could not be investigated in this study. Although this is less distinct, our results indicate that in the Walloon Region the association between daily alcohol drinking and obesity has changed over time. Surprisingly, daily drinking alcohol seems to have a positive impact on obesity. The value of this indicator is however limited as the quantity of alcohol consumed was not taken into account.

The National Health Interview Survey is a powerful tool due to its horizontal approach of data collection: information on the health status (i.e. BMI), socio-demographic background characteristics and lifestyle factors are collected at the same time for the same person. Furthermore, it includes a large sample of the general population [37]. Because participation in this survey is not compulsory, the response rate is rather low. This problem was experienced in all the survey years [13]. The survey method and most of the questions have remained relatively unchanged over time. Even though the questions related to alcohol drinking and the daily consumption of fruit and vegetables are an exception to this rule, as they slightly changed between 1997 and 2008, it is assumed that this had no impact on the principal findings of our research. A possible artefact could be associated to the question concerning leisure time physical activity which in the first two surveys was included in the face-to-face questionnaire and later moved to the self-reported questionnaire. If this is the case, the artifact would have been identified equally throughout the regions.

However, some shortcomings of the data should be highlighted. The indicator ‘eating daily fruit and vegetables’ is the only one of the food pallet. Other eating habits, such as fat or sugar consumption, could have a bigger influence on bodyweight. Unfortunately the number of food frequency questions included in the survey is limited, resulting in a lack of details about the dietary habits. The nutritional indicator that we used has several facets which can also be applied for other lifestyle factors. Our choice of the lifestyle indicators is partly based on the availability of the data from the four surveyed years. The choice of these indicators could have an influence on the study results. In addition, self-reported questions on behaviors such as alcohol consumption, smoking, diet and physical habits are subject to a social desirability; the prevalence of a lack of leisure time physical activity is probably underestimated. A more reliable method to measure physical activity would have been to rely on accelerometers, but this technique is expensive and therefore not applicable to large population surveys [38]. In summary, it is possible that the available lifestyle data inadequately reflected the lifestyle dimensions that had an impact on the different regional time trends of obesity.

Although the BMI is a valuable tool to provide a standardized definition of obesity for the purposes of national surveillance and international comparisons [39], it has some drawbacks, especially when height and weight are self-reported. Studies have highlighted that when calculating the BMI via self-reported data, obesity will be underestimated because overweight participants tend to understate their weight and all participants tend to over-report their height [2,5,8,9]. Underestimation of obesity is also related to the fact that obese people more often refuse to take part in surveys [1]. Moreover, the use of the BMI does not take into account the body fatness and body fat distribution which are associated with obesity comorbidities [8,9,20]. Although the BMI measurement through self-reported data is biased, it is unlikely that this bias substantially changes over time. Consequently the assumption that the evolution of obesity via self-reported BMI yields reasonable valid results is quite plausible.

Current physical activity levels in the three Belgian regions are still far too low to efficiently counterbalance the prevalence of obesity. Even though the study is unable to link the campaigns to promote physical activity to the prevention of obesity, these campaigns have existed during the studied period and differed by region (in Belgium health promotion and prevention is primarily dealt with at the regional level). In the period 2002–2006, the Flemish Region launched a campaign with the main purpose to strengthen self-esteem (feeling good as you are). The second wave of this campaign focused on healthy and active lifestyle, to improve the quality of life (2003) and on healthy nutrition (2004). Physical activity was a specific target only in 2003. After 2008, the Flemish Region dedicated its campaign to improve physical activity, along with healthy nutrition. It will consequently be interesting to verify the results of the next health survey conducted in 2013. On the other hand, the campaign in the Walloon and the Brussels Regions during 2005–2010 was promoting healthy habits, including both nutrition and physical activity. This campaign was launched after the health survey of 2004.

Epidemiological research has shown clear health gains through staying active, which has already been understood by scientists and academics, but not by the policy makers. Promoting physical activity by the governments is still far less a priority than that of other lifestyle factors, such as smoking and nutrition. Encouraging people to be physically active has numerous benefits that go beyond weight control. A number of psychological benefits have been identified, with the clearest being depression and anxiety [40]. Obesity can be prevented with lifestyle changes involving increased physical activity [1,3,5,7,14,18,19]. Promoting physical activity has therefore to remain a priority. Regular and adequate levels of physical activity – at least an hour a day – is important to keep a steady weight, or even reduce it, and to fight the obesity epidemic. Consequently, a healthy weight will have a positive effect on the health outcomes [23,41].

## Conclusion

The decreasing association between obesity and a lack of leisure time physical activity in the Walloon Region between 1997 and 2008 could indicate that there is an increasing awareness of risk factors for obesity in the Walloon population, which may have resulted in a more favourable evolution of the obesity epidemic in Wallonia than in the two other regions.

## Competing interests

The authors declared that they have no competing interests.

## Authors’ contribution

SDr drafted the paper. JVdH and SD helped with the interpretation of the data. JVdH, SD and JT reviewed and commended the manuscript. All authors approved the final and submitted version.
